# A Survey on Unmanned Underwater Vehicles: Challenges, Enabling Technologies, and Future Research Directions

**DOI:** 10.3390/s23177321

**Published:** 2023-08-22

**Authors:** Arif Wibisono, Md. Jalil Piran, Hyoung-Kyu Song, Byung Moo Lee

**Affiliations:** 1 Department of Intelligent Mechatronics Engineering and Convergence Engineering for Intelligent Drone, Sejong University, Seoul 05006, Republic of Korea; arifwibisono@sju.ac.kr; 2 Department of Computer Science and Engineering, Sejong University, Seoul 05006, Republic of Korea; piran@sejong.ac.kr; 3 Department of Information and Communication Engineering and Convergence Engineering for Intelligent Drone, Sejong University, Seoul 05006, Republic of Korea; songhk@sejong.ac.kr

**Keywords:** unmanned underwater vehicle, propulsion and dive system, sensing, localization, energy resources and supply, thorp model, USV–UAV–UUV joint-design operation

## Abstract

Unmanned underwater vehicles (UUVs) are becoming increasingly important for a variety of applications, including ocean exploration, mine detection, and military surveillance. This paper aims to provide a comprehensive examination of the technologies that enable the operation of UUVs. We begin by introducing various types of unmanned vehicles capable of functioning in diverse environments. Subsequently, we delve into the underlying technologies necessary for unmanned vehicles operating in underwater environments. These technologies encompass communication, propulsion, dive systems, control systems, sensing, localization, energy resources, and supply. We also address general technical approaches and research contributions within this domain. Furthermore, we present a comprehensive overview of related work, survey methodologies employed, research inquiries, statistical trends, relevant keywords, and supporting articles that substantiate both broad and specific assertions. Expanding on this, we provide a detailed and coherent explanation of the operational framework of UUVs and their corresponding supporting technologies, with an emphasis on technical descriptions. We then evaluate the existing gaps in the performance of supporting technologies and explore the recent challenges associated with implementing the Thorp model for the distribution of shared resources, specifically in communication and energy domains. We also address the joint design of operations involving unmanned surface vehicles (USVs), unmanned aerial vehicles (UAVs), and UUVs, which necessitate collaborative research endeavors to accomplish mission objectives. This analysis highlights the need for future research efforts in these areas. Finally, we outline several critical research questions that warrant exploration in future studies.

## 1. Introduction

Unmanned vehicles are developed to operate in various environments, including those operating on the surface of the water, known as unmanned surface vehicles (USVs) [1], those operating in the air as unmanned aerial vehicles (UAVs) [2], and those that operate underwater as unmanned underwater vehicles (UUVs). Currently, UUVs are growing in communication, control systems, and automation, and are even using Machine Learning processes such as setting trajectories, sensing, and managing flocks of unmanned vehicles [3].

As compared to unmanned vehicles that operate on the ground and those that operate in the air, which are able to access wireless communication systems properly, the conditions for unmanned vehicles that operate on the ground are different. This is due to the reliability of air in transmitting communication signals, especially with relay technology and shared resource management [4]. In spite of the fact that there are several disturbances, fault handling, recovery, and fault management can still be used to handle them [5]. Meanwhile, UUVs are affected by fluid properties, which interfere with signal propagation [6]. Furthermore, the energy required to transmit the signal is relatively high [7], whereas the signal received is lower, resulting in the loss of large data [6,8].

The underwater communication technologies can be categorized into five models: (1) acoustic communication that uses sound waves as a communication signal [9]; (2) optical communication uses visible and invisible light waves [10]; (3) wireless communication via radio waves [11]; (4) Satellite communication communicates with devices in the water through intermediary relays on the surface [12]; (5) direct electrical communication used for UUV charging docks [13,14].

There are four indicators that can be used to measure communication effectiveness: bit-error-rate (BER); signal-to-noise ratio (SNR) in decibels (dB); spectral efficiency, which is decrypted as the number of bits per second that can be transmitted through a specific bandwidth unit (bps/Hz); and energy efficiency. Specific propagation loss coefficient and noise floor are employed for different signal frequencies, as well as water characteristics (such as temperature, salinity, and depth) [6]. The signal-to-noise ratio measures the strength of the signal compared to the background noise. Better communication conditions are indicated by a higher SNR.

The propulsion system and dive system allow the UUV to move and control its depth in the water [15]. To propel the vehicle through the viscosity of water, one or more thrusters are used [16,17]. The propulsion system also controls the vehicle’s buoyancy, usually using a ballast system, which absorbs or releases water to regulate the vehicle’s buoyancy, as well as control systems to control dives and ascents [18]. UUVs can also be propelled by wings, fins, and hydrojets [19,20,21,22].

Five types of UUVs are considered in the control system, which focuses on control algorithms: the first is proportional–integral–derivative (PID) [23], which utilizes sensor feedback and processes it proportionally, integrally, and derivatively. Integrational means accumulating the errors over time and adjusting the control output based on those errors, while proportional means comparing; for example, comparing the output with the desired output. Derivative means calculating the error rate and adjusting the output based on that rate. The second type, model predictive control (MPC), predicts the future behavior of the system [24]. It uses a mathematical technique called sliding mode to achieve robust control of a system, regardless of variations in its dynamics or disturbances in the environment. Sliding mode control or SMC is a third method [25]. Fourth, adaptive control (AC) uses a mathematical model of the system to estimate the current state of the system [26] and then adjusts the control inputs accordingly. Last but not least, adjusting UUV trajectory [27] or UUV herd localization uses Artificial Intelligence and Machine Learning (ML) [28]. Control algorithms are chosen based on the application and the requirements given, but there are a number of testing models that can be used, including (1) simulation; (2) closed-loop testing; (3) comparison with other algorithms; and (4) measuring key performance indicators (KPIs).

UUV uses several sensor devices in environmental sensing and recognition systems, and they are grouped based on how they work into four types: acoustic-sensors, such as sound navigation and ranging (SONAR) [29] and hydrophones [30], that detect and locate objects in water using sound waves; optical sensors, such as cameras and light detection and ranging (LiDAR) [31,32], that use light waves to capture images and gather data on the surface of the water; chemical-sensors [33], which can detect and measure the concentration of dissolved gases, pollutants, and other substances in the water [34]; and physical-sensors [35], which can detect and measure the properties of water and its surroundings. Using these four types of sensors together can provide a comprehensive picture of the underwater environment for navigation, object detection, and environmental monitoring. As a result of the sensing function, UUV operations can be conducted to collect various types of data, such as bathymetry [36], water quality [32], images of the seafloor [32], etc., depending on the mission and the sensors and instruments installed on the vehicle.

Several types of navigation systems can be used by unmanned vehicles underwater, including inertial navigation systems (INS) which use accelerometers and gyroscope sensors to measure linear and angular motion, and once the angular position and orientation are known, the location of the UUV can be estimated [37,38]. Another application using similar measurement data is the Doppler velocity log (DVL), which measures the relative velocity of UUV to water by using the theory of the Doppler effect [39,40,41,42]. Furthermore, the third system is the use of the global navigation satellite system (GNSS) and global positioning system (GPS) which utilize satellite signals to determine UUV position and speed [43] based on the difference between a signal transmitted by a satellite and a signal received by a GPS receiver. In addition, the GNSS system transmits longitude, latitude, altitude and time signals simultaneously [44,45,46]. Currently GPS/GNSS technology can only be used for vehicles operating in the sea surface environment, such as the Vessel UUV, and further research is needed in order to apply it to the underwater environment. Furthermore, the fourth system introduced in this paper is the use of sound waves to determine the position and speed of the UUV using an acoustic navigation system (ANS) [47]. Acoustic signals can be measured by measuring the time delay or the Doppler shift [48]. To track the position and orientation of the UUV, the last or fifth system is the use of visual odometry (VO) [49]. UUV localization optimization can be improved by integrating navigation systems, various sensors, and other data sources, as mentioned above. In summary, the key point is that GNSS can provide absolute position measurements, while INS and DVL can provide accurate speed measurements. This integration is referred to as sensor fusion, i.e., when sensors and data sources are integrated in one unit.

The primary source of energy for most unmanned underwater vehicle is batteries [50]. Depending on the specific application and mission requirements, these batteries can be rechargeable or disposable. UUVs have also used fuel cells [51], hydrogen [52], and solar panels [53], which require cooperative control systems to harvest solar energy continuously if the energy source battery backup is low. The UUV’s energy requirements and supply will be determined by the specific mission and operating conditions, such as mission duration, water depth, and payload type.

Additionally, several studies predict an increase in demand for underwater vehicles in the future [54,55]. The underwater wireless sensor network (UWSN) [56] infrastructure is also being utilized to support the continuity of operation of the UUV which has limitations when operating in water. Because it is supported by air–water boundary communication technology (AWBC) [57,58] and underwater cyber physical system (UCPS) for data collection needs [59,60], it is possible to collaborate on communication and control systems [57,58] for multi-environmental unmanned vehicles. Internet of Underwater Things or (IoUT) localization techniques based on reinforcement learning [61] can be used in collaboration with vessel-based UUVs to collect marine survey information [62]. Several other studies, such as those based on controversy adjudication (CATM) [63], are expected to further improve the effectiveness of UUV operations underwater and eliminate its limitations [64].

While the poor supporting technology in the underwater environment has implications for many things, including difficulties controlling the UUV, reduced situational awareness, difficulty executing missions, and the risk of losing the UUV [54], reliable supporting technology is urgently needed. Furthermore, the entire discussion outlined above opens this survey paper and invites readers to better understand the concept of unmanned vehicles operating underwater, as well as the importance of technological support.

This paper employs state-of-the-art writing, which flows and makes it easy for readers to read, including an introduction, which provides information about unmanned vehicles, their types, communication technology, propulsion and dive systems, control systems, sensors, localization, energy resources, and supplies that support the operation of UUVs under water. It explains the workings of the supporting technology from a technical and a mathematical perspective. We also explain how the literacy survey was conducted, starting with frequently asked research questions, statistical trends, and the keywords used to find articles that support the general and specific statements.

An integrated mathematical approach is then used to discuss the work system of each UUV-supporting technology, including communication technology, propulsion, control systems, sensing, localization, and energy resources. Next, through simulations, the performance of technology supporting UUV operations is simulated. Then, we examine the performance gaps found in research in supporting technology performance, discussing the latest issues such as the implementation of the Thorp model for the distribution of shared resources for communication and energy, as well as a joint-design of USV–UAV–UUV operations for completing a mission that requires future research contributions. Finally, we outline several critical open research challenges for future studies.

The paper is organized as follows: an introduction that covers types of unmanned vehicles, UUV support technology, research contributions, and the state of the art. This is followed by a discussion of related works, including research questions, existing surveys, and statistical trends. Next, a coherent and mathematical approach is used to discuss the UUV work system and conduct a performance simulation. The paper concludes with a discussion on future research directions and a summary of the performance gaps found.

Recent research advances in the field of underwater vehicles encompass various aspects, including sensing, cross-boundary cooperation patterns of autonomous vehicles, optimization of cross-boundary communication utilizing signal propagation theories within water and signal modulation engineering, the adoption of successful models from autonomous vehicle types operating in terrestrial-aerial environments, and the incorporation of deep learning and deterministic Artificial Intelligence technologies. Collectively, these aspects represent future research avenues that can be developed based on what the researcher has presented in this review.

**Figure 1:**
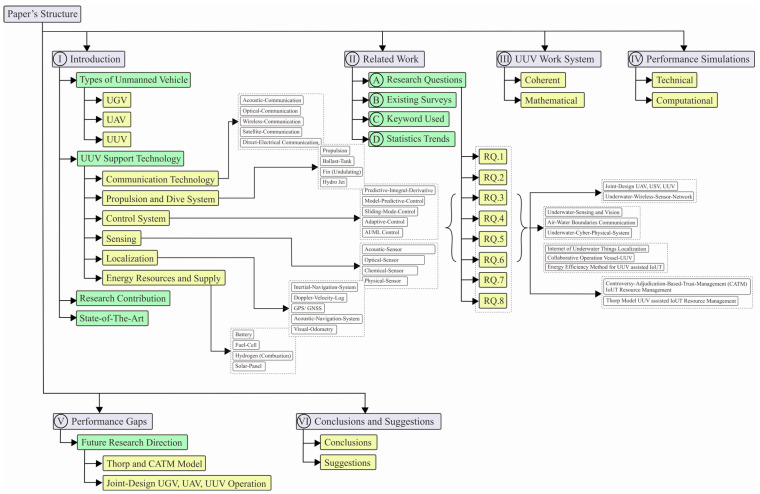
Organization of the paper.

Figure 1 shows the structure of this survey paper more detail.

## 2. Related Works

In addition to using a correlational method, this survey considers literacy novelty. In order to perform this task, we follow the following steps.

1. Identifying research topics by considering the need for survey contributions, summarizing questions that are frequently asked in similar surveys, etc.;
2. Examining similar survey papers to identify subtopics that have not been reviewed;
3. Searching for answers using general and specific keywords;
4. Identifying future research directions by looking at trends statistically.

## 3. Research Questions

In this field of research, we are motivated to explore and answer some frequently asked questions (FAQ). These questions are clearly summarized in Table 1.

### 3.1. Existing Surveys

By comparing FAQs with answers, we provide a summary of statements in other research articles that answer the questions raised, which can be seen clearly in Table 2.

### 3.2. Keyword Used

**Figure 2:**
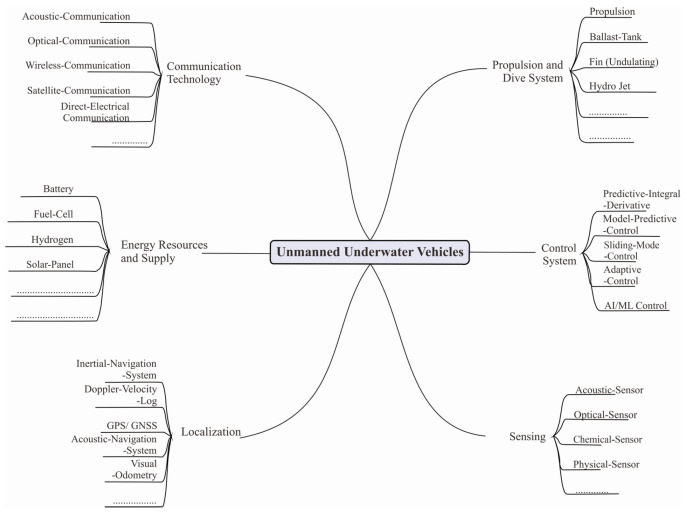
Survey taxonomy based on general to specific keywords.

As part of this research, general and specific keywords are used to locate supportive references, compile mindmaps, compare studies, search for appropriate research approach models, examine future trends, and determine what contribution is needed in this area of research. References used have a publication year limit of 2017–2023 with a minimum citation level of 2 and are from reputable journals. Keywords are used to identify research directions and support general theoretical and technical statements. For technical discussions, special formulations, and simulations, special keywords are used. As shown in Figure 2, the search results based on general and specific keywords in the field of UUV and its supporting technology are illustrated in a branching graph.

### 3.3. Statistics Trends

We classify acronyms used as unique identifiers for several studies using search engines and reference management applications. To understand trends in this field of research, we also identify publications by the publisher in the second table. The grouping data is shown in Table 3 and Table 4:

## 4. UUV Work System

A mission scenario illustrates how an unmanned vehicle works in an underwater environment.

### 4.1. Underwater Commmunication

In communication systems that use the hexagonal model for localization (buoy) as a ground station transmitter relay, the hexagonal model represents the sphere-shaped Earth, which is mathematically divisible by the hexagonal model. Relay placement by measuring the effective beacon distance is calculated as follows [67]:

(1) $$C=\frac{\cup_{i=1}^{N}V_{n}\left(i\right)}{V},$$

Network coverage ratio is represented by *C*, summazation volume monitoring area is denoted by *V*, and monitoring area of node $V_{n}\left(i\right)$ is denoted as $n_{i}$. It is assumed that the nodes are equipped with an omidirectional antenna that monitors in all directions (sphere area) which has a radius and is denoted in $r_{s}$.

The signal transmission system uses a combination of acoustic communication for long-distance transmission at sea depth [9], optical for fast short-range communication in the depths of the sea [10], and radio waves for communication at sea level between the USV, relay station, and ground transmitting station [11]. Additionally, the USV is equipped with a direct electrical [13,14] system that allows for the recharging of UUV swarms operating underwater and a swarm drone carrier with an air–water boundary communication system [29]. Repeating the previous statement that a higher SNR ratio indicates better communication conditions, to calculate the SNR value [29], we proceed with the following:

(2) $$SNR=\frac{{\left(I_{r}\right)}^{2}}{\sigma_{n}^{2}},$$

where $I_{r}$ is the received light intensity and $\sigma_{n}^{2}$ is the variance of noise within the system. $I_{r}$ can be represented as

(3) $$I_{r}=I\cdot L_{t}\cdot L_{ch}\cdot A\cdot I_{s}\cos\psi .$$

In the context of the study, the following terms are defined: *I* represents turbulence-induced channel fading, following a lognormal distribution; $L_{t}$ and $L_{ch}$ denote temperature and channel loss, respectively; $I_{s}$ represents irradiance in the pattern of ideal emission; ψ is the incident angle of the receiving plane; and *A* represents the active area of the photodiode.

(4) $$\sigma_{n}^{2}=2qℜP_{n}B$$

In the given context, the following terms are defined: *q* is the charge of an electron; $P_{n}$ represents the solar noise power, *B* denotes the signal bandwidth; and *ℜ* is the responsivity of the photodiode.

### 4.2. Dive System

A herd of UUVs in the mission scenario consists of three types, each distinguished by its propulsion and dive system. The first UUV is propelled by propellers on all sides, referred to as omnidirectional [15,16,17], and is able to move in any direction to maximize its efficiency. The Buoyancy force generated by the object expressed in newton (N), $\Delta_{Bouyancy}$ can be represented as:

(5) $$\Delta_{Bouyancy}=\pi \times \rho_{water}\times \frac{{\left(D_{out}\right)}^{2}}{4}\times L,$$

where π is the mathematical constant pi, approximately equal to 3.14159; $\rho_{water}$ is the density of water expressed in (kg/m^3^); $D_{out}$ is the outer diameter of the submerged object expressed in meters (m); and *L* is the length of the submerged object expressed in meters (m).

The origin axis of the vehicle is located at the middle of the *x*–*y* axis and at the bottom of the *z*-axis. To calculate the center of gravity with respect to the origin axis, the following formula can be used:

(6) $$\begin{array}{c} M_{x}=\sum_{i=0}^{i=n}W_{i}\chi X_{i}+W_{ai}\chi X_{ai} \end{array}$$

(7) $$\begin{array}{c} M_{y}=\sum_{i=0}^{i=n}W_{i}\chi Y_{i}+W_{ai}\chi Y_{ai} \end{array}$$

(8) $$\begin{array}{c} M_{z}=\sum_{i=0}^{i=n}W_{i}\chi Z_{i}+W_{ai}\chi Z_{ai}. \end{array}$$

**Figure 3:**
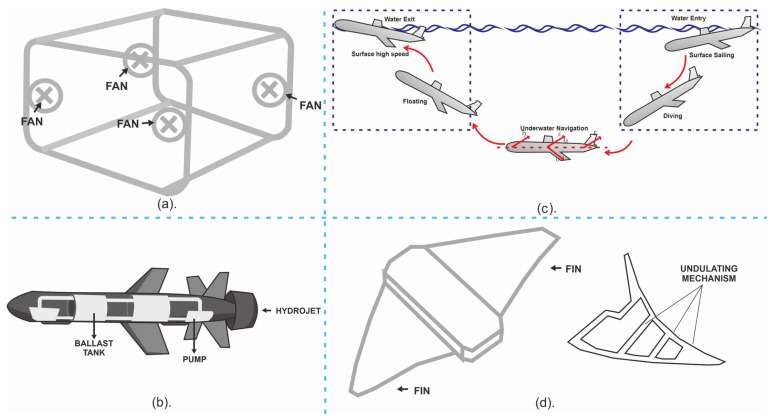
(**a**) Omnidirectional, (**b**) hydrojet, (**c**) hydrojet maneuver and (**d**) undulating propulsion and dive system [16,21,22].

The following terms are defined in the context of the study: $W_{i}$, $W_{ai}$, $X_{i}$, $Y_{i}$, $Z_{i}$, and $X_{ai}$, $Y_{ai}$, $Z_{ai}$ represent the weight component, added weight by ballast water, offset of the center of weight component, and added weight component, respectively. For UUVs that utilize an omnidirectional dive system, a visual representation of the system can be observed in Figure 3a.

The second type of UUV uses a hydrojet propulsion system and diving system [22]. Known as a cross-domain vehicle (CDV), this vehicle can operate in both surface and underwater environments because it is equipped with a ballast tank, propulsion system, and rudder. The buoyancy law [68] is used in this approach, which is based on:

(9) $$\Delta B=(F_{B}-F_{G})/g=M-\nabla \rho .$$

In the given context, the following terms are defined: ΔB represents the net buoyancy in kg; $F_{G}$ denotes the gravitational force; $F_{B}$ represents the buoyant force exerted by the fluid on the floating object; *M* is the total mass of the object; ∇ denotes the volume of the fluid displaced by the object; and ρ is the density of the fluid. The rudder is responsible for directing the CDV into three modes of motion, as outlined below.

1. Dynamic model of surface state:
  (10) $$\begin{array}{cc} mU_{i}= & P\cos\theta -F\cos(\alpha -\theta )\\ \; & -F_{f}\cos\left(\beta -\theta \right)-F_{r}\cos\left(\gamma -\theta \right), \end{array}$$
  (11) $$\begin{array}{c} mU_{j}=F\sin\left(\alpha -\theta \right)+F_{f}\sin\left(\beta -\theta \right)\\ +F_{r}\sin\left(\gamma -\theta \right)+P\sin\theta -G, \end{array}$$
  (12) $$J_{k}\theta =M+F_{f}l_{f}\sin\beta -F_{r}l_{r}\sin\gamma .$$
2. Dynamic model of the underwater state:
  (13) $$\begin{array}{cc} mU_{i}= & P\cos\theta -F\cos(\alpha +\theta )\\ \; & -F_{f}\cos\left(\beta -\theta \right)-F_{r}\cos\left(\gamma -\theta \right), \end{array}$$
  (14) $$\begin{array}{cc} mU_{j}= & F_{v}\cos\theta +F_{f}\sin\left(\beta -\theta \right)+F_{r}\sin\left(\gamma -\theta \right)\\ \; & +P\sin\theta +G-F\sin(\alpha +\theta ), \end{array}$$
  (15) $$J_{k}\theta =F_{v}l_{v}+F_{f}l_{f}\sin\beta -F_{r}l_{r}\sin\gamma -M.$$
3. Underwater and surface transition state:
  (16) $$\begin{array}{cc} mU_{i}= & P\cos\theta -F\cos\left(\alpha -\theta \right)-F_{f}\cos\left(\beta -\theta \right)\\ \; & -F_{r}\cos\left(\gamma -\theta \right)-F_{v}\sin\theta , \end{array}$$
  (17) $$\begin{array}{cc} mU_{j}= & F_{v}\cos\theta +F_{f}\sin\left(\beta -\theta \right)+F_{r}\sin\left(\gamma -\theta \right)\\ +P\sin\theta +F\sin(\alpha +\theta )-G, \end{array}$$
  (18) $$J_{k}\theta =F_{v}l_{v}+F_{f}l_{f}\sin\beta +M-F_{r}l_{r}\sin\gamma .$$

Table 5 provides detailed information regarding each symbol and unit. An illustration of a UUV that uses a propulsion system and hydrojet diving is shown in Figure 3b, while Figure 3c illustrates movement maneuvers resulting from the three equations above.

**Figure 4:**
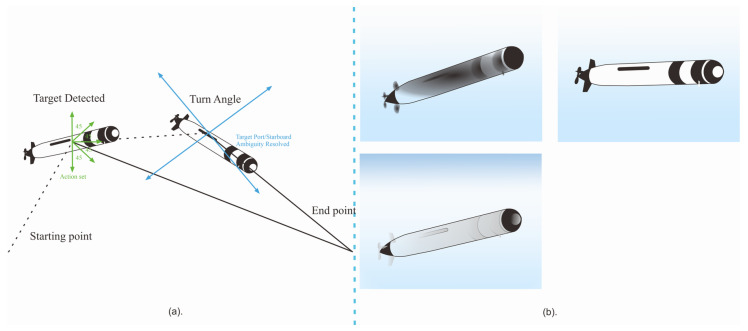
Sensing tracking target involves a sequential process depicted in (**a**). Initially, upon target detection, the measured aspect angle remains unresolved, as evidenced by both port and starboard rays extending from the vehicle. Subsequent maneuvering and data collection during the second leg facilitate the completion of the resolve-ps-ambiguity behavior, leading to the determination of target orientation. Once resolved, the keep-broadside behavior utilizes the obtained vehicle-relative bearing measurement, indicated by a single ray extending from the vehicle, to effectively track the target’s movements. (**b**) In the realm of optical sensing, a progression unfolds from the top left to right, followed by a downward transition. The initial view showcases the original image captured. Subsequently, the HSV image processing unveils a distinct perspective, enabling the extraction of key color information. Finally, the journey culminates in the presentation of color masks, representing specific regions of interest and aiding in targeted analysis. This holistic sensing approach harmonizes various stages of processing, culminating in a comprehensive understanding of the tracked target’s dynamics and optical properties [30,34].

Futhermore, a third type of UUV is the undulating UUV; this means that the UUV is equipped with fins that function simultaneously as a propulsion and diving system. In their study, the authors provided a comprehensive account of the intricate six-degrees-of-freedom (DOF) motion exhibited by the manta ray robot [21]. This encompassing movement involves a spectrum of displacements including longitudinal, sideways, and vertical shifts, in addition to the nuanced roll, pitch, and yaw rotations. The manta ray robot’s overall motion finds representation through elegant flowing vectors: $\eta ={\left[x,y,z,\varphi ,\theta ,\psi \right]}^{T}$, $v={\left[u,v,w,p,q,r\right]}^{T}$, and $\tau ={\left[X,Y,Z,K,M,N\right]}^{T}$. Here, η captures the vector describing the robot’s position and attitude in the earth-fixed frame, while *v* characterizes the body-fixed linear and angular velocity vector. The intricate interplay of forces and moments acting on the robot within the body-fixed frame is succinctly described by τ, as depicted in Figure 4. By assuming the robot’s movement takes place in an ideal fluid at a consistent velocity of $V_{0}$ and by disregarding the effects of water’s viscosity and inertia, the equations governing the robot’s motion are elegantly simplified, particularly in the vertical plane.

(19) $$\begin{array}{cc} mu= & \left(G-B\right)\sin\theta -F_{X} \end{array}$$

(20) $$\begin{array}{cc} mw= & \left(G-B\right)\cos\theta -F_{Z} \end{array}$$

(21) $$\begin{array}{cc} B= & B_{0}+\Delta B \end{array}$$

(22) $$\begin{array}{cc} G= & G_{0}+\Delta G \end{array}$$

(23) $$\begin{array}{cc} F_{X}= & \frac{1}{2}\rho u^{2}S_{x}C_{x} \end{array}$$

(24) $$\begin{array}{cc} F_{Z}= & \frac{1}{2}\rho w^{2}S_{y}C_{y} \end{array}$$

The intricate interplay of forces and factors influencing the manta ray robot’s behavior is encapsulated by a set of key variables. These include *G*, representing the robot’s gravitational force; $G_{0}$, which captures the robot’s gravity in the absence of the mass block; and ΔG, signifying the gravitational impact of the mass block itself. Furthermore, the buoyant forces at play are delineated by *B*, indicating general buoyancy; $B_{0}$, reflecting the buoyancy when equated to the robot’s gravity; and ΔB, representing an adjustable buoyancy component. Within this fluid dynamic context, $F_{X}$ and $F_{Z}$ step forward as vital descriptors of fluid resistances acting along the *x*-axis. The fundamental physical attributes of the robot and its environment are given voice by *m* denoting the robot’s mass and ρ standing for the water density. The geometric characteristics of the robot are encapsulated by $S_{x}$ and $S_{y}$, the maximum transverse and longitudinal cross-sectional areas, while the nuanced hydrodynamics are unveiled by $C_{x}$ and $C_{y}$, the hydrodynamic parameters that contribute to the robot’s interaction with its surroundings. Each of these variables weaves together to define the intricate dance of forces and dynamics that shape the manta ray robot’s journey. Refer to Figure 3d for a visualization of the propulsion and undulating diving system.

### 4.3. Control

The first drone used a model-predictive-control algorithm to set the maneuvers and trajectory of the herd UUV, which predicts future behavior and optimizes a control action based on that prediction. According to Saback et al. [24] and Heshmati-Alamdari et al. [68], whose logic flow employs a discrete-time form:

(25) $$x_{k+1}=f\left(x_{k},\tau_{k}\right)\Rightarrow x_{k}+1=x_{k}+\Lambda \left(x_{k}\right)dt+∁\left(\tau_{k}\right)dt,$$

where

(26) $$\Lambda \left(x_{k}\right)=\left[\begin{array}{c} u_{rk}\cos\left(\psi_{k}\right)-v_{rk}\sin\left(\psi_{k}\right)+u_{c}^{\tau }\\ u_{rk}\sin\left(\psi_{k}\right)+v_{rk}\cos\left(\psi_{k}\right)+v_{c}^{\tau }\\ w_{rk}+w_{c}^{\tau }\\ r_{rk}\\ \frac{1}{m_{11}}(m_{22}v_{rk}r_{rk}+X_{u}u_{rk}+X_{u|u|}\left|u_{rk}\right|u_{rk})\\ \frac{1}{m_{22}}(-m_{11}u_{rk}r_{rk}+Y_{v}v_{rk}+Y_{v|v|}\left|v_{rk}\right|v_{rk})\\ \frac{1}{m_{33}}(Z_{w}w_{rk}-m_{22}u_{rk}v_{rk}+Z_{w|w|}\left|w_{rk}\right|w_{rk})\\ \frac{1}{m_{44}}(\left(m_{11}-m_{22}\right)u_{rk}v_{rk}+N_{r}r_{rk})\\ +N_{r|r|}\left|r_{rk}\right|r_{rk} \end{array}\right]$$

(27) $$∁\left(\tau_{k}\right)=\left[\begin{array}{c} 0_{4}\times 1\\ T_{A}\tau_{k} \end{array}\right].$$

$x_{k}={\left[\eta_{k}^{T},v_{rk}^{T}\right]}^{T}\in R^{8}$ represents the state vector at time step *k*, which includes the position and orientation of the vehicle with respect to the inertial frame τ, and the relative linear and angular velocity of the vehicle with respect to the water. $m_{11},i=1,\ldots ,4$, $X_{u},Y_{v},Z_{w},N_{r}<0$, $X_{u|u|},Y_{v|v|},Z_{w|w|},N_{r|r|}>0$, and $d_{t}$ denote the mass terms, linear, and quadratic drag terms, and sampling period, respectively. The control input of the system is $\tau_{k}={\left[\tau_{pk},\tau_{sk},\tau_{vk},\tau_{lk}\right]}^{T}\in R^{4}$, representing the thrusters’ forces. Ocean current profile uncertainties are presented by $\delta_{k}={\left[0_{1\times 4},\delta_{uk},\delta_{vk},\delta_{wk},\delta_{rk}\right]}^{T}\in D\subset R^{8}$, with *D* being a compact set and $\left|\right|\delta_{k}\left|\right|\leq \delta$. The perturbed system is modeled by taking into consideration the disturbances caused by ocean current profile uncertainties and dynamic parameter uncertainties denoted by $\Delta f(x_{k},\tau_{k})$. The vehicle’s dynamic parameters are assumed to have been identified through a proper identification scheme.

(28) $$\begin{array}{cc} x_{k+1}= & f\left(x_{k},\tau_{k}\right)+\delta_{k}\\ \; & =f\left(x_{k},\tau_{k}\right)+\Delta f\left(x_{k},\tau_{k}\right)+\delta_{k}\\ \; & =f\left(x_{k},\tau_{k}\right)+\gamma_{k}+\delta_{k} \end{array}$$

where

(29) $$\begin{array}{c} \gamma_{k}=\Delta f\left(x_{k},\tau_{k}\right)\in T,\\ \left|\right|\gamma_{k}\left|\right|\leq \gamma \forall x_{k}\in X,\\ \tau_{k}\in T, \end{array}$$

where *T* is the compact set of uncertainties bounded by γ≥0.

(30) $$x_{k+1}=f\left(x_{k},\tau_{k}\right)+w_{k}$$

Let $w_{k}=\gamma_{k}+\delta_{k}\in W\subset R^{8}$ be the vector of uncertainties and external disturbances affecting the system. *W* is a compact set, defined as W=D⊕T, where *D* and *T* are also compact sets. Hence, *W* is bounded by $\left|\right|w_{k}\left|\right|\leq w$, where $w\overset{\Delta }{=}y+\delta$. The dynamical equation of the system includes the vector of disturbances. However, for the nominal model, we neglect the effect of disturbances.

Employing a sliding-mode control algorithm, the second drone distinguishes itself through the implementation of a nonlinear control strategy. This strategy, characterized by its simplified logical framework, stands resilient against various disturbances and uncertainties that might arise during operation. The foundation of this innovative approach is rooted in the insightful work of Qiao et al., who have introduced a significant advancement in trajectory tracking control. Referred to as the fast integral terminal sliding mode control (FITSM) method, it represents a refined iteration of the ITSM method, as discussed in their authoritative references [25]. This amalgamation of cutting-edge techniques underscores the second drone’s prowess in achieving precise and robust control, poised to navigate challenges with a balanced blend of sophistication and adaptability.

(31) $$s\left(t\right)=e\left(t\right)+\alpha e_{I}\left(t\right),$$

(32) $$e_{I}\left(t\right)=e^{m/n}\left(t\right),\;\mathrm{with}\;e_{I}\left(0\right)=-\alpha^{-1}e\left(0\right).$$

In the context of this control framework, let us denote e(t)∈R as the tracking error, where its significance cannot be understated. To further shape the dynamics, a positive constant α is introduced, playing a pivotal role in influencing the system’s behavior. Additionally, we introduce the integers *m* and *n*, both of which are odd, with a clear constraint ensuring that *n* holds a greater value than *m*, and both remain greater than zero. The interplay of these elements intertwines to orchestrate a controlled system marked by intricate relationships and carefully orchestrated dynamics.

In the scenario where s(t) remains consistently at zero, effectively dictating that e(t) takes on the form $-\alpha e_{I}\left(t\right)$, a fascinating consequence unfolds within the realm of the fractional integrator. Under these conditions, the fractional integrator demonstrates its distinctive behavior and characteristics, showcasing the remarkable interplay between the components involved. This alignment not only offers insights into the system’s response but also unveils a unique facet of the fractional integration process that emerges when specific constraints are meticulously maintained.

(33) $$e_{I}\left(t\right)=-\alpha^{m/n}e_{I}^{m/n}\left(t\right)$$

The solution to the error dynamic provides crucial insights into the behavior and evolution of the system, unraveling the intricate interplay of variables and shedding light on its underlying dynamics.

(34) $$e_{I}\left(t\right)={\left[e_{I}^{1-m/n}\left(0\right)-\alpha^{m/n}\left(1-m/n\right)t\right]}^{1/(1-m/n)}$$

Subsequently, the time at which $e_{I}\left(t\right)$ achieves convergence is determined, yielding a fundamental understanding of the temporal aspect of this critical variable’s behavior.

(35) $$t_{r}=\frac{{\left|e_{I}\left(0\right)\right|}^{1-m/n}}{\alpha^{m/n}\left(1-m/n\right)}=\frac{{\left|e(0)\right|}^{1-m/n}}{\alpha (1-m/n)}$$

The convergence of the tracking error e(t) to the ITSM surface s(t)=0 is achieved within a finite timeframe under the condition $e\left(t\right)=-\alpha e_{I}\left(t\right)$. This pivotal observation encapsulates the essence of the FITSM approach, characterizing it as a dynamic system where the interplay of variables culminates in this precise convergence scenario.

(36) $$s\left(t\right)=e\left(t\right)+\alpha e_{I}\left(t\right)$$

(37) $${\dot{e}}_{I}\left(t\right)=e\left(t\right)+\beta e^{m/n}\left(t\right),\;\mathrm{with}\;e_{I}\left(0\right)=-\alpha^{-1}e\left(0\right)$$

On the FITSM surface, s(t)=0 (i.e., $e\left(t\right)=-\alpha e_{I}\left(t\right)$), with β>1 and other parameters defined as in the ITSM. The integrator is equivalent to

(38) $${\dot{e}}_{I}\left(t\right)=-\alpha e_{I}\left(t\right)-\alpha^{m/n}\beta e_{I}^{m/n}\left(t\right).$$

We adapt Chu et al.’s approach presented in [69] for developing a nervous system-based control system for AUVs using DRL-based control. This control system transforms local environmental information into an array $S_{1}=\left(s_{1},s_{2}\right)$, where $s_{1}$ represents the direction of the ocean current.

(39) $$s_{1}={\left[\begin{array}{ccc} X & \cdots & X\\ \vdots & \ddots & \vdots \\ X & \cdots & X \end{array}\right]}_{n\times n,}$$

where, X∈[0,360] and $s_{1}$ represent the ocean current direction, while $s_{2}$ is a matrix with information on obstacles and ocean currents. To ensure AUV safety, we define a forbidden area around each obstacle considering changing ocean current directions and eddy currents.

(40) $$s_{2}={\left[\begin{array}{cccccc} 3 & \cdots & \cdots & 3 & 3 & 3\\ 3 & \ddots & 2 & 2 & 2 & 3\\ \vdots & \ddots & 1 & 1 & \ddots & \vdots \\ \vdots & \ddots & 1 & 1 & \ddots & \vdots \\ \vdots & \ddots & \ddots & \ddots & \ddots & \vdots \\ 1 & \cdots & 3 & 1 & 2 & 1 \end{array}\right]}_{n\times n,}$$

where “1” represents the obstacle area; “2” indicates the prohibited zone; and “3” is the navigation region. The navigation state vector, $S_{2}=\left(\vartheta_{1},s_{1},s_{2},\upsilon_{1}\right)$, represents the angle between vector $\vec{\alpha }$ and $\vec{\beta }$, where $\vec{\alpha }$ and $\vec{\beta }$ depict the points from the current and initial locations to the destination, respectively. This crucial value can be acquired through the following method:

(41) $$\vartheta_{1}=\cos^{-1}\frac{\vec{\alpha }\cdot \vec{\beta }}{\left|\vec{\alpha }\right|\cdot \left|\vec{\beta }\right|}.$$

The allocation of the destination within the AUV coordinate system is succinctly represented by the direction variables $\left(s_{1},s_{2}\right)$, encapsulating the spatial arrangement of the target point. This system is meticulously defined as follows:

(42) $$\left(s_{1},s_{2}\right)=\left\{\begin{array}{ccc} \left(0,0\right) & \; & \mathrm{The}\;\mathrm{first}\;\mathrm{quadrant}\\ \left(0,1\right) & \; & \mathrm{The}\;\mathrm{second}\;\mathrm{quadrant}\\ \left(1,0\right) & \; & \mathrm{The}\;\mathrm{third}\;\mathrm{quadrant}\\ \left(1,1\right) & \; & \mathrm{The}\;\mathrm{fourth}\;\mathrm{quadrant}. \end{array}\right.$$

### 4.4. Sensing

The underwater unmanned vehicle (UUV) is outfitted with an array of sensors, which encompass passive, active, or fused sensing capabilities, enabling it to perceive the surrounding environment, underwater entities, and fellow UUVs. As detailed in [30], precision in measurements is attainable solely when the target resides within the sonar’s effective field of view. Upon acquiring a set of *N* measurements during the initial leg, the UUV’s behavior dictates both its operational mode and the corresponding turn angle δ∈[0,π], calculated through the utilization of $\bar{\alpha }$. Operating under a non-preferential turning direction, the vehicle consistently executes right turns, expressed in radians as the turn angle unfolds.

(43) $$\delta \left(\bar{\alpha }\right)=\left\{\begin{array}{cc} \pi /2, & \mathrm{if}\;\bar{\alpha }\in \left[0,\omega_{1}\right)\cup \left(\pi -\omega_{1},\pi \right]\\ \pi /2, & \;\mathrm{if}\;\bar{\alpha }\in \left[\omega_{1},\omega_{2}\right)\cup \left(\pi -\omega_{2},\pi -\omega_{1}\right]\\ \bar{\alpha }-\omega_{1}, & \mathrm{if}\;\bar{\alpha }\in \left[\omega_{2},\pi -\omega_{2}\right] \end{array}\right.$$

The array configuration follows a structured pattern: the initial row corresponds to the first leg, the subsequent row pertains to the second leg, and the final row is designated for the broadside target. This systematic arrangement effectively organizes the data collected. To visualize the operational process of the system, refer to Figure 4a, which visually captures the step-by-step functioning of the system.

Song et al. [34] suggested using a color screening filter based on hue–saturation value (HSV) to detect oil leaks. However, RGB color space, which is commonly used in optical displays, is not accurate enough for oil spill segmentation. In HSV space, the video can be converted to screen the oil spill region under the foreground mask. The computation methodologies for the *S* and *V* channels are well-defined: *S* is determined as v−min(R,G,B), while *V* is calculated as max(R,G,B). Equally integral is the calculation of the *H* channel, which follows the subsequent process:

(44) $$H=\left\{\begin{array}{c} 60(G-B)/(V-\min(R,G,B))\;\mathrm{if}\;V\;=\;R\\ 60(B-R)/(V-\min(R,G,B))\;+\;120\;\mathrm{if}\;V\;=\;G\\ 60(R-G)/(V-\min(R,G,B))\;+\;240\;\mathrm{if}\;V\;=\;B. \end{array}\right.$$

By applying the threshold screening process to the HSV model, areas suspected of being affected by oil spills can be described effectively using the following equation:

(45) $$Mask_{i}=\left\{\begin{array}{cc} 1, & {\underline{T}}_{i}\leq I_{i}\leq {\bar{T}}_{i}\\ 0, & \mathrm{others}. \end{array}\right.$$

The process involves setting lower threshold ${\underline{T}}_{i}$ and upper threshold ${\bar{T}}_{i}$ values for each color channel. These thresholds are applied to individual pixel values, denoted as $I_{i}$, within the HSV color space. The resultant mask pixel value is assigned as 1 if and only if the pixel values across all three channels satisfy the threshold conditions; otherwise, it is assigned a value of 0. For a more comprehensive understanding of the HSV concept, refer to Figure 4b, which provides a visual elucidation of the HSV model’s intricacies. This visual aid serves to enhance clarity in grasping the nuances of HSV-based thresholding.

Employing the HSV extraction method within the realm of autonomous intelligence for autonomous underwater vehicles (AUVs) holds profound significance. This technique empowers AUVs to not only detect but also comprehend intricate color details present within their aquatic surroundings. Such an ability proves indispensable for the AUVs’ capacity to conduct thorough and insightful analyses of the underwater environment, thereby enhancing their overall capabilities and contributions to underwater exploration and research. AUV can detect objects based on distinctive color patterns, understand its surroundings, aid in navigation and obstacle avoidance, and enhance underwater observation and monitoring. Machine learning techniques can also be used for color-based object recognition, identifying environmental changes, and predicting environmental conditions. The development of autonomous intelligence through the combination of HSV extraction and Machine Learning holds the potential to improve AUV’s adaptability and interaction with the underwater environment, enhancing AUV’s mission performance in various applications, such as resource exploration, marine environmental monitoring, and scientific research beneath the ocean’s surface. More about the potential use of Machine Learning in AUV operations is discussed in the future research directions chapter.

### 4.5. Localization

We use two models for UUV localization. The first model, adapted from Braginsky et al. [42], uses the Doppler velocity log (DVL) method. DVL sends out acoustic beams and measures the Doppler frequency shift to compute the velocity and direction of each beam. Relevant definitions and calculations are as follows: $R_{b}^{n}$ is the rotation matrix defined by Euler angles (ϕ,θ,ψ).

(46) $$\begin{array}{cc} R_{b}^{n}\left(\phi ,\theta ,\psi \right) & \\ \; & =\left[\begin{array}{ccc} c\theta c\psi & s\phi s\theta c\psi -c\phi s\psi & c\phi s\theta c\psi +s\phi s\psi \\ c\theta s\psi & s\phi s\theta s\psi +c\phi c\psi & c\phi s\theta s\psi -s\phi c\psi \\ -s\theta & s\phi c\theta & c\phi c\theta \end{array}\right] \end{array}$$

Using the DVL method for UUV localization involves two models. The first sends out acoustic beams and measures the Doppler frequency shift for each beam to compute velocity and direction. To transform a vector from body-fixed to DVL coordinates, we use the following coordinate transformation with cα=cos(α) and s(α). Correction of DVL measurement requires considering seafloor-to-platform angles during velocity calculations. Assuming the local seafloor is represented by the plane equation $z_{i}=a+bx_{i}+cy_{i}$ for i=[1⋯4] representing the DVL’s four altitude measurements, the measurement can be expressed in matrix form.

(47) $$z=A{\left[\begin{array}{ccc} a & b & c \end{array}\right]}^{T}.$$

**Figure 5:**
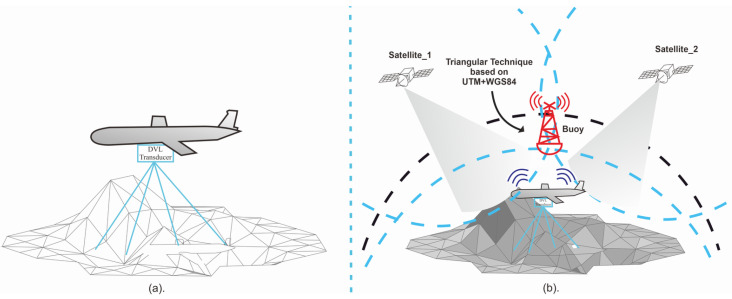
(**a**) Two-dimensional seafloor example. The blue line represents the DVL signal that performs seafloor measurements and estimates. (**b**) GPS–GNSS Work [42].

Using the seafloor equation and linear algebra, we estimate angles $\hat{\phi }$ and ${\hat{\theta }}_{s}$. Figure 5a provides an illustration of the DVL and its measurement.

We are demonstrating GPS- or GNSS-based location techniques for unmanned surface vehicles (USV). However, GPS measurements in water are inaccurate and misleading. According to Jiang et al. [43], GPS works on the principle of 2D Cartesian coordinates (x,y) and their respective covariances, using *Universal Transverse Mercator (UTM)* from the *World Geodetic System (WGS84)* ellipsoid. This theory is illustrated in Figure 5b.

Measurement by GPS $z_{t}^{GPS}$ provides the position and orientation parameters written in the equation:

(48) $$\begin{array}{cc} z_{t}^{\;\mathrm{GPS}}= & \langle \left[\begin{array}{c} \mu^{\;\mathrm{GPS}}\\ \mu_{\theta }^{\;\mathrm{GPS}} \end{array}\right],\;\left[\begin{array}{cc} \Sigma^{\;\mathrm{GPS}} & 0\\ 0 & \sigma_{\theta }^{\;\mathrm{GPS}} \end{array}\right]\rangle ,\\ \mu^{\;\mathrm{GPS}}= & \left[\begin{array}{c} \mu_{x}^{\;\mathrm{GPS}}\\ \mu_{y}^{\;\mathrm{GPS}} \end{array}\right],\;\Sigma^{\;\mathrm{GPS}}=\left[\begin{array}{cc} \sigma_{x}^{{\;\mathrm{GPS}}^{2}} & \sigma_{xy}^{\;\mathrm{GPS}}\\ \sigma_{xy}^{\;\mathrm{GPS}} & \sigma_{y}^{{\;\mathrm{GPS}}^{2}} \end{array}\right]. \end{array}$$

Assuming that position (x,y) and orientation (θ) follow a Gaussian probability density function (PDF), the posterior given a GPS reading can be obtained according to the following:

(49) $$p\left(x_{t}|z_{t}^{\mathrm{GPS}}\right)\;∼p\left(z_{t}^{\mathrm{GPS}}|x_{t}\right)\;\cdot p\left(x_{t}\right)\;=\;\;f\left(x,y\right)\;\cdot \;f_{\mathrm{WN}}\left(\theta \right)\;\cdot \;p\left(x_{t}\right),\;\;\;\;\;$$

where the PDF for the position is in

(50) $$f\left(x,y\right)\;=\;\frac{1}{2\pi \;\;\;{\left|\Sigma^{\mathrm{GPS}}\;\right|}^{1/2}}e^{-\frac{1}{2}\left({\left[\begin{array}{c} x\;-\;\mu_{x}^{\mathrm{GPS}}\\ y\;-\;\mu_{y}^{\mathrm{GPS}} \end{array}\right]}^{T}\Sigma^{{\mathrm{GPS}}^{-1}}\left[\begin{array}{c} x\;-\;\mu_{x}^{\mathrm{GPS}}\\ y\;-\;\mu_{y}^{\mathrm{GPS}} \end{array}\right]\right)},\;\;\;\;\;$$

and the PDF corresponding to the orientation angle, which follows a wrapped normal distribution:

(51) $$f_{\mathrm{WN}}\left(\theta \right)\;=\;\frac{1}{\sigma_{\theta }^{\mathrm{GPS}}\sqrt{2\pi }}\sum_{k=-\infty }^{\infty }e^{-{\left(\theta -\mu_{\theta }^{\mathrm{GPS}}\;+\;2\pi k\right)}^{2}/2\sigma_{\theta }^{{\mathrm{GPS}}^{2}}}.$$

### 4.6. Energy Supply

In addition to internal battery power, UUVs can utilize potential renewable energy sources in the aquatic environment. Baik et al. [51], Sezgin et al. [52], and Tian et al. [53] have explored this topic, and we summarize their findings in Table 6.

Lindsay et al. [59] and Fang et al. [63,64] suggest using track lines and a collaborative approach with unmanned underwater vehicles and systemized underwater communication resources to improve energy efficiency during underwater survey missions.

We propose using UAVs equipped with reconfigurable intelligence surface (RIS) devices [70,71] as communication relays to expand coverage to previously unreachable water areas.

**Figure 6:**
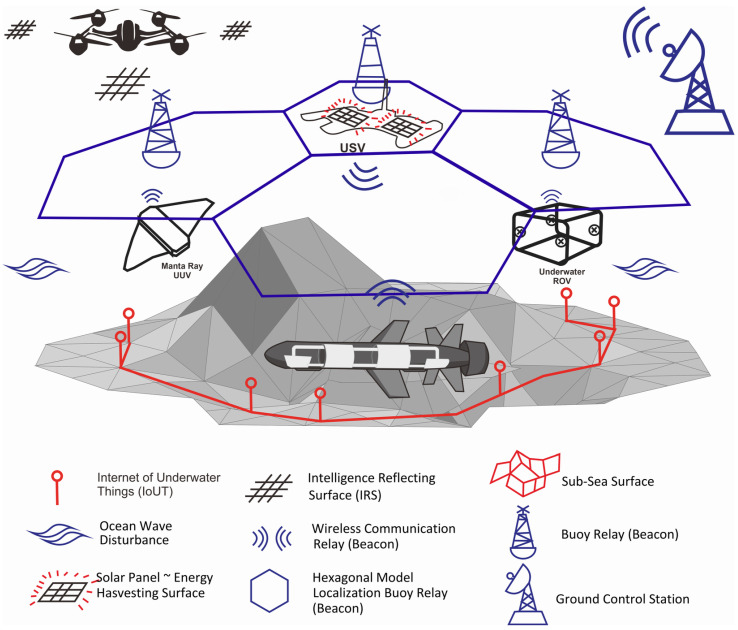
Scenario joint-operation: UAV, USV, UUV.

The joint operation scenario for UAV, USV, and UUV is shown in Figure 6 and summarized in Table 7.

In a series of sequentially arranged mission illustrations, we can form a comprehensive overview of potential collaboration patterns in the execution of missions involving various types of unmanned vehicles (Unmanned Aerial Vehicle (UAV); Unmanned Surface Vehicle (USV); and Unmanned Underwater Vehicle (UUV)). This concept is supported by networking and communication resources operating both underwater and on the surface. This collaborative approach integrates diverse unmanned vehicle platforms and holds the potential to revolutionize cross-domain mission execution.

Previous research references have discussed the benefits of each type of unmanned vehicle separately within the contexts of maritime and aerial missions. UAVs prove valuable for aerial monitoring and data collection, USVs are suitable for surface water monitoring and patrols, and UUVs can perform exploration and reconnaissance in underwater depths. However, to optimize these potentials, the integration of these elements into a coordinated framework is necessary.

In the described scenario, cooperation between UAVs, USVs, and UUVs is facilitated by a robust network and communication infrastructure in both the air and water environments. This enables these vehicles to share real-time information, coordinate movements, and execute complementary tasks across various environmental layers. For instance, UAVs can gather data from the air and transmit them to USVs on the surface, which can then direct UUVs to conduct further surveys in the ocean depths.

The application of this concept holds extensive potential, ranging from environmental monitoring missions, military reconnaissance, and exploration of marine resources to disaster response in maritime settings. By harnessing the strengths of various types of unmanned vehicles and the support of cross-air and water communication networks, we can create an adaptable, responsive, and efficient system for cross-domain missions. In conclusion, through the amalgamation of ideas from various preceding research references, we have delineated a comprehensive vision of how collaboration among unmanned vehicles, supported by cross-air and water communication networks, can shape new working paradigms in solving cross-domain missions. This concept harbors substantial potential for optimizing resource utilization, expanding operational scope, and delivering innovative solutions to diverse challenges in both maritime and aerial environments.

## 5. Performance Simulations

The previous section described a swarm of UUVs and their six leading supporting technologies: underwater communication; dive system; control; sensing; localization; and energy supply. These technologies are computationally simulated to estimate malfunctions and performance gaps.

### 5.1. Underwater Communication

In the introduction to this paper, we stated our aim to achieve a minimum BER and maximum SNR for the communication system [6]. Direct communication is ideal for achieving this, while other methods such as acoustic communication have a long transmission time [72], optical communication has a short range [10,73], and wireless radio and satellite communications experience high attenuation in water.

We assumed that wired communication is the best alternative with low SNR and BER. However, it lacks flexibility. Optical communication is the second-best option with low SNR and BER, but it has limited range. On the other hand, the most equitable and feasible alternative for use is the acoustic communication model, which is directly transmitted underwater. Wireless and satellite communications perform poorly because they still require intermediary media to connect between the aerial and underwater domains.

### 5.2. Dive System

**Figure 7:**
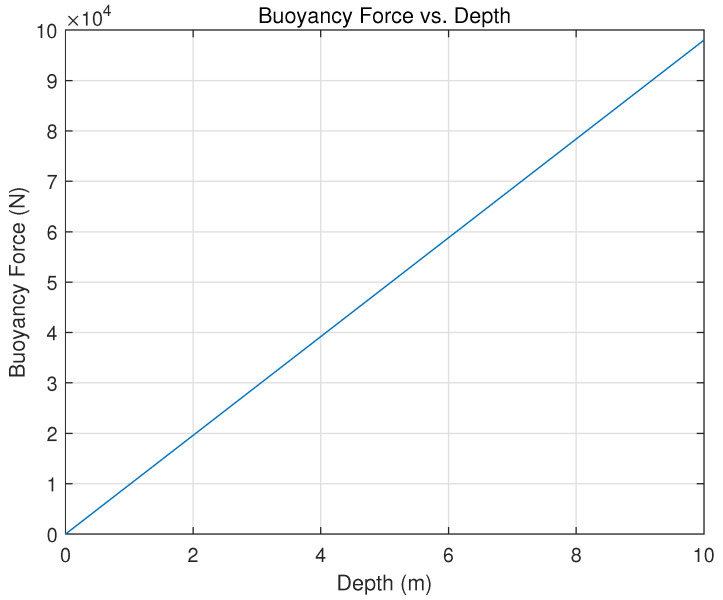
Simulation of a diving system using the law of buoyancy: $\delta_{i}=\left(i_{t}W-i_{b}W\right)$ approach, where $\delta_{i}$ is a coefficient attitude-dependent magnitude of the moment produced by any offset between the center of mass ($i_{t}$) and buoyancy ($i_{b}$) and (*W*) is the weight of the vehicle.

A diving system’s performance is measured by synchronizing the required energy with the resulting buoyancy. It uses the $\delta_{i}=\left(i_{t}W-i_{b}W\right)$ approach, where $\delta_{i}$ is a coefficient attitude-dependent magnitude of the moment produced by any offset between the center of mass ($i_{t}$) and buoyancy ($i_{b}$), and (*W*) is the weight of the vehicle [15,19]. This statement is supported by the simulation results shown in Figure 7.

### 5.3. Control

**Figure 8:**
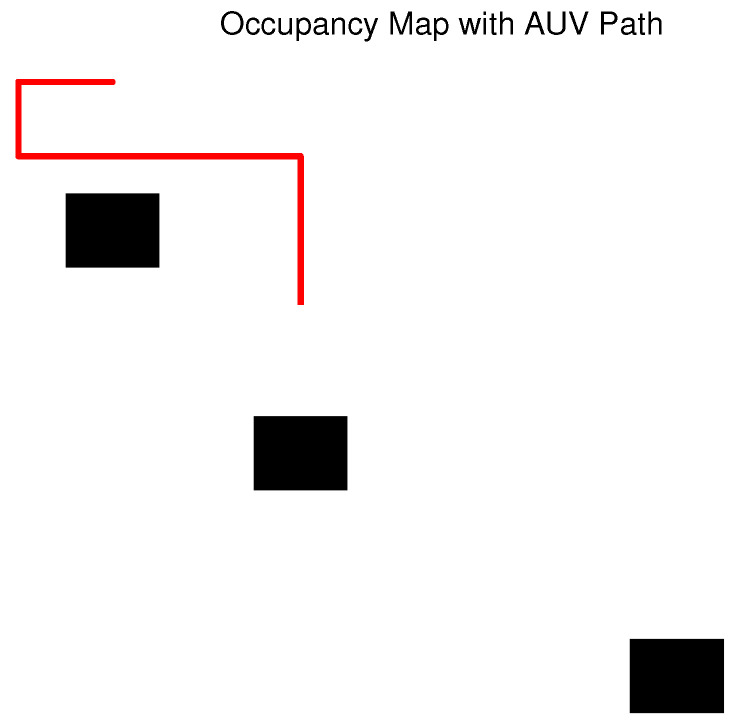
Control system: these waypoints, represented by a vector $p_{k}$, can be written in the equation $\omega_{k}=\left[x_{pk},y_{pk},z_{pk},V_{pk}\right]$, where $x_{pk}$, $y_{pk}$, and $z_{pk}$ are the absolute coordinates of the waypoints in the environment frame. $V_{pk}$ is the desired norm of the AUV velocity vector (mostly surge and dive) at the considered waypoint (can be 0).

The accuracy of the control system can be measured by the unmanned vehicle’s ability to follow a predetermined path using Cartesian coordinates (X,Y,Z). The path-following controller’s goal is to enable autonomous navigation through a series of waypoints represented by a vector $p_{k}$, defined by the equation $\omega_{k}=\left[x_{pk},y_{pk},z_{pk},V_{pk}\right]$. The KPIs for the control system are $V_{pk}$, which are shown in the simulation results in Figure 8.

### 5.4. Sensing

**Figure 9:**
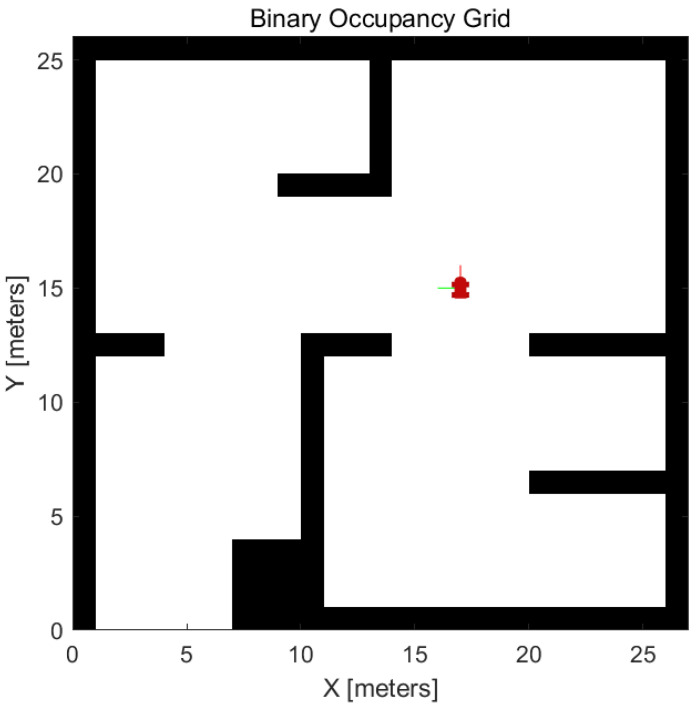
Sensing: works by changing the environment image into light–dark parameters, where the light path (1 $I_{i}\leq {\bar{T}}_{i}$) means it can be skipped and the dark band (0 ${\underline{T}}_{i}\leq I_{i}$) must be avoided.

The UUV sensing performance, simulated as in Song et al. [35], converts environmental parameters into hue–saturation value (HSV) images for easy recognition via binary computing systems. The HSV threshold values ${\underline{T}}_{i}$ and ${\bar{T}}_{i}$ are set for each color channel, and the pixel value on the mask is 1 only when the pixel values on all three channels meet the threshold requirements. This is illustrated in Figure 9.

### 5.5. Localization

**Figure 10:**
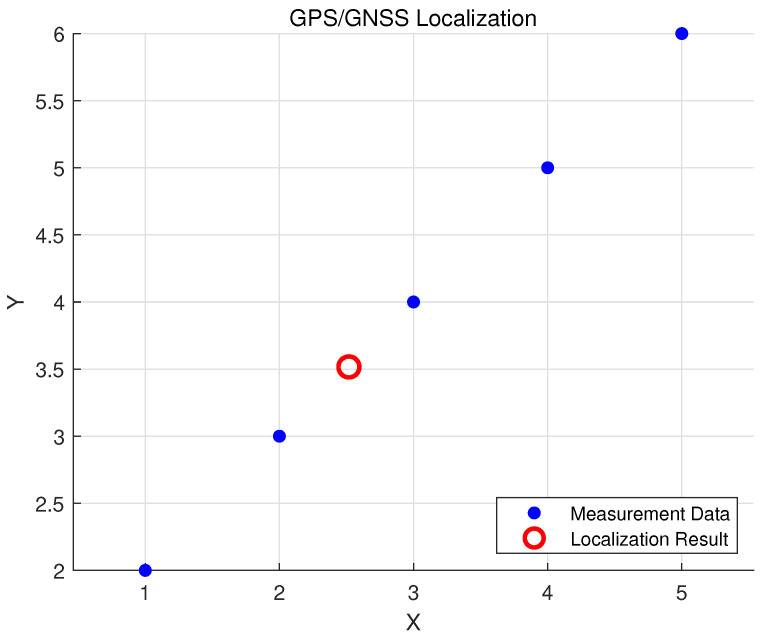
Localization: works by identifying (x,y) position and (θ) as orientation.

Localization performance is indicated by the ability to identify UUV position and orientation [43], with the GPS reading represented as $p\left(x_{t}|z_{t}^{GPS}\right)!∼p\left(z_{t}^{GPS}|x_{t}\right)\cdot p\left(x_{t}\right)=f\left(x,y\right)\cdot f_{WN}\left(\theta \right)\cdot p\left(x_{t}\right)$. This is supported by simulation results in Figure 10.

### 5.6. Energy Supply

**Figure 11:**
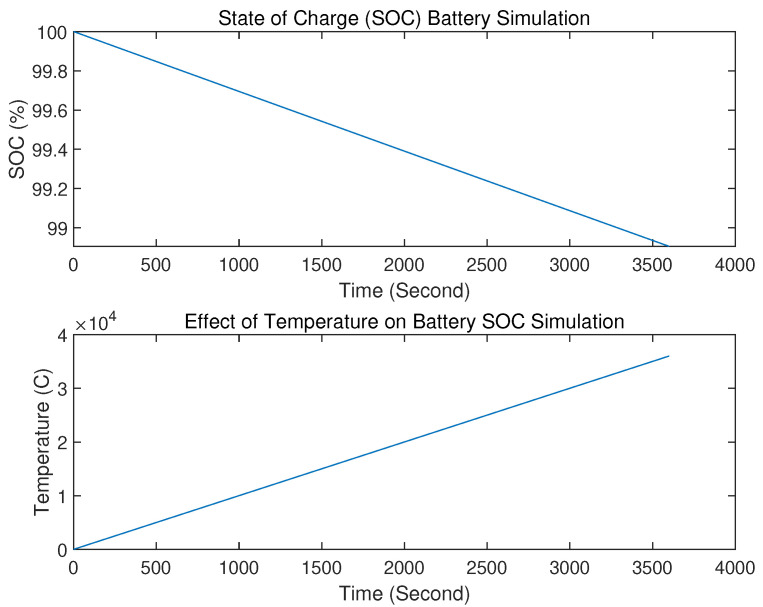
Energy supply: battery state of charge (SoC), which is affected by ambient temperature, where $P_{load}=\left(Re\left(Z_{r0}\right)\right)I_{p}^{2}=\omega_{0}\frac{M^{2}}{L_{s}}I_{P}^{2}Q_{s}\cdot T$.

UUV battery performance depends on ambient temperature during charging. Low temperatures can reduce capacity, while high temperatures can increase battery aging and shorten lifespan. The equation $P_{load}=\left(Re\left(Z_{r0}\right)\right)I_{p}^{2}=\omega_{0}\frac{M^{2}}{L_{s}}I_{P}^{2}Q_{s}$ (Teeneti et al. [15]) can be modified by adding temperature (T), as $P_{load}=\left(Re\left(Z_{r0}\right)\right)I_{p}^{2}=\omega_{0}\frac{M^{2}}{L_{s}}I_{P}^{2}Q_{s}\cdot T$. Figure 11 shows the optimal temperature range for storage capacity and lifespan.

## 6. Performance Gaps

This research takes a general approach to the field of underwater vehicles and communications with the aim of identifying gaps for further investigation.

1. Currently, underwater communication technology lacks a description of actual underwater signal conditions and instead relies on calculated approaches using existing research and surveys.
2. We only simulate buoyancy and its energy requirements, without making comparisons.
3. We have only measured UUV control system effectiveness based on time. However, we have not compared models using other parameters such as system autonomy or algorithm capabilities during system failure. Additionally, the optimal control algorithm should trace the shortest path based on our assumptions.
4. We use the HSV method to convert environmental parameters into computer-readable notations of 0 and 1. However, due to the complex underwater environment and its impact on sensing functions, more research is necessary to identify additional parameters.
5. Underwater localization technology only tracks GPS locations, identifying (x,y) position and θ orientation. Real-time, accurate positions of underwater vehicles require consideration of factors such as speed and orientation, whether diving or floating. Combining sensor functions to form an IMU and calculate position with GPS is also worth considering. Therefore, further research on this topic is needed.
6. Simulations provided an overview of the ambient temperature’s effect on the vehicle’s state of charge capability for energy supply. However, further simulations are necessary, including battery life calculations, as underwater vehicles operate remotely and system failures due to power outages can be challenging to evacuate. Therefore, more research is required on this topic.

## 7. Future Research Directions

The study analyzes the performance gap between UUV operation support technology and the latest research in the field. Further research on resource management, including communication, dive system, control, sensing, localization, and energy supply, is being considered. The Throp model approach, adapted from Menaka et al. [74] and the CATM model by Jiang et al. [65], is used to optimize UUV operational capabilities using energy network infrastructure resources, communication, and underwater environment monitoring. Menaka [74] emphasizes the importance of resource management in communication and underwater vehicle research, which will become the backbone of future sea-related research. The study by Jiang et al. [65] supports this claim. Mathematical and computer simulations are conducted using IoUT-assisted underwater communication and sensing resource-sharing management.

In future research, we will propose joint operations of AUV, UUV, and USV, optimize the air–water boundary communication model [29], and model the use of reconfigurable intelligence surfaces (RIS) in AUVs to support these joint operations [71].

In the implementation of UUV operations, it is possible that Doppler effect may occur, similar to what happens in mobile cellular communication. This is due to the changes in the location and distance between the transmitter (TX) and receiver (RX) as the UUV moves relative to such changes. The extent of the impact of this can be calculated using basic mathematical approaches:

(52) $$f_{D}=\left(\frac{v\pm v_{d}}{v\pm v_{s}}\right)f_{S},$$

where $f_{D}$ is the detected frequency; $f_{S}$ is the frequency emitted by the source; *v* is the speed of sound waves through the underwater environment; $v_{D}$ is the relative detector speed with respect to the underwater environment; and $v_{S}$ is the speed of sound waves. A simple illustration is that there is a stronger frequency change as the source approaches the detector, and the opposite occurs as the source moves away. The values of the other parameters will automatically adjust to this phenomenon.

Some studies have anticipated this phenomenon and offered solutions, such as Li et al. [75] who also discuss the Doppler shift phenomenon in underwater acoustic (UWA) communication and propose a multicarrier orthogonal frequency division multiplexing (OFDM) communication system to overcome this challenge. Furthermore, Abdelkareem et al. [76] offer a Doppler shift compensation scheme by modifying the carrier frequency of OFDM subcarriers to match the Doppler shift frequency. In the study by Li et al. [75], an experiment was conducted using different numbers of subcarriers—512, 1024, and 2048—each with the corresponding number of active subcarriers: 484, 968, and 1936, respectively. The experiment focused on bit rates, employing a fixed guard interval of $T_{g}=25$ and an OFDM block duration *T* representing values of 42.8, 85.38, and 170.73, respectively. For these setups, the bit rates without coding resulted in 10.52 kb/s, 12.90 kb/s, and 14.55 kb/s. Furthermore, after applying a rate 2/3 channel coding, the bit rates were observed to 7.0 kb/s, 8.6 kb/s, and 9.7 kb/s, respectively [75].

From the observation above, it can be concluded that the working mechanism of OFDM multicarrier is capable of addressing the classic issue of Doppler shift, as indicated by the constant interval. However, there are certain concerns related to the limitations of this approach. Therefore, an examination of the Doppler effect, its implications, and strategies by which to mitigate it should also be included as part of future research directions in the field of unmanned underwater vehicles.

In addition to future issues regarding the technologies that can be integrated into AUV operations, there are also concerns about potential applications that may arise in the future given the rapid and advancing field of research. We predict that with the development of communication technology and the increasing demands in underwater vehicle research for various purposes such as resource exploration, marine environmental monitoring, and scientific research, there may also be a demand for research in the field of maritime transportation assisted by underwater vehicles, autonomous surface vehicles, and the utilization of Artificial Intelligence technology. Due to the limited references on the utilization of underwater vehicles, autonomous surface vehicles, and Artificial Intelligence technology, we have drawn some references from similar research fields applied to different devices and environments, such as the use of autonomous aerial vehicles in some case studies, including unmanned transportation options equipped with cognitive awareness capabilities that enable UAVs to actively recognize and understand their surroundings, making smarter and more responsive decisions in various situations, and studies using UAVs for autonomous traffic monitoring and management assisted by Artificial Intelligence.

Among the researchers examining and presenting their findings are Filippone et al. [77], who discussed the developments in urban air mobility and the use of rotorcraft as air transportation options in urban areas. Furthermore, Barmpounakis et al. [78] conducted a review on the application of unmanned aerial vehicle systems in transportation engineering, covering current practices and future challenges. Additionally, Cavaliere et al. [79] researched the development of proactive Unmanned Aerial Vehicles (UAVs) to enhance cognitive contextual awareness, aiming to integrate Artificial Intelligence and data processing technologies to enable UAVs to actively recognize and understand their surroundings, making smarter and more responsive decisions in various situations, including obstacle avoidance and mission adaptation based on environmental changes. Vlahogianni et al. [80] conducted research on model-free traffic condition identification using unmanned aerial vehicles (UAVs) and deep learning, developing a data processing model for traffic analysis from aerial perspectives and training deep learning algorithms to recognize traffic density patterns. Moreover, Trivedi et al. [81] developed a real-time vision-based vehicle detection and speed measurement system using morphology and binary logical operations. The research aims to create a method capable of accurately detecting vehicles in real time using visual data from cameras and accurately measuring vehicle speeds based on inter-frame movement, achieved by combining morphology and binary logical operation techniques.

If successfully implemented in terrestrial and aerial environments, the possibility of applying these techniques to surface and underwater environments in the case of autonomous underwater vehicles may also be feasible. However, researchers must also address factors that could lead to system and operational failures, considering the unique underwater environment distinct from other environments.

Furthermore, the development of applying Machine Learning for optimization across various sectors involving computation is also predicted to have an impact on the research and development of unmanned underwater vehicles. Several studies supporting this statement include Teng et al. [82], who investigated underwater target recognition methods based on deep learning frameworks (DL); Bhopale et al. [83], who developed obstacle avoidance systems based on reinforcement learning (RL) for autonomous underwater vehicles (AUVs); and Sands and Timothy [84], who developed deterministic Artificial Intelligence (AI) for unmanned underwater vehicles, where “deterministic” signifies that decisions made by the AI system possess a high degree of certainty and definiteness. This leverages predictability and consistency in the behavior of the AI system, implying that it produces identical responses or actions when confronted with the same situation.

Research by Sun et al. [85] involved developing a three-dimensional path tracking control system for autonomous underwater vehicles using a deep reinforcement learning (DRL) approach. Another study by Sun et al. [86] focused on mapless motion planning systems for AUVs using a policy gradient-based DRL approach. The main emphasis of policy gradient-based methods is on learning policies that link environmental states to actions that need to be taken by the agent. A notable advantage of this approach is its capability to address continuous action spaces and exhibit stochastic (probabilistic) policies.

From the entire series of reviews, several proposals can be summarized which are expected to be able to overcome common problems and issues regarding research in the field of unmanned underwater vehicles, among others shown in Table 8.

## 8. Conclusions

From the overall discussion above, several conclusions can be drawn by the researchers.

First, the prospects for the research and development of autonomous underwater vehicles (AUVs) in the future heavily rely on technological support. Collaborative communication technology capable of overcoming the limitations of signal transmission across air and water or signal transmission at water depths, utilizing support from the underwater communication network infrastructure, is crucial. It is essential for UUVs to have energy-efficient propulsion systems, sustainable power supply, reliable navigation control, and robust sensing capabilities, as the success of missions depends on these technological supports, as demonstrated in several simulations where efficiency and optimization are the primary focus of attention.

Second, resource management can enhance efficiency, and with good resource management supported by available infrastructure, joint operations involving AUVs, USVs, and UUVs can be deployed simultaneously.

Third, future research is likely to focus on implementing new technologies that can potentially be integrated to address research barriers and challenges. The application of autonomous underwater vehicles similar to autonomous vehicles operating on land and in the air can be considered by exploring new theories and utilizing the currently available technological support, including underwater communication technology and Artificial Intelligence.

## Figures and Tables

**Table 1:** FAQ about UUV surveys and their supporting technologies.

S. No	Related Research Questions	Answer
RQ1	How can UUVs be used to collect data?	Depending on the mission and the sensors and instruments installed on the vehicle, UUVs can collect a variety of data such as bathymetry, water quality, imagery of the seafloor, and other types [28,31,32,36,41,42,49].
RQ2	How do UUVs navigate and control their movements in water?	To move through water, UUVs use navigation and control systems. An inertial navigation system, a GPS system, and a sonar system are a few examples [20,21,23,28,37,43,44,54,55,59].
RQ3	What are the ways in which UUVs communicate and store data?	UUVs have wireless communication systems for transmitting data and storage devices for storing data, such as hard drives or solid-state drives [6,29,31,59,65].
RQ4	How do UUVs obtain power?	Alternative energy sources such as fuel cells, batteries, and lithium-ion batteries are used to power UUVs [54,62,63,66].
RQ5	How can UUVs be equipped with payloads?	UUVs can be equipped with various payloads to perform specific tasks such as sampling, imaging, and mapping [23,28,32,44,49,54,55,59].
RQ6	What are the steps involved in planning and controlling a UUV survey?	In order to plan and control their missions, UUVs and UAVs use mission planning and control software. The software can be used for navigation, sensor control, and data analysis [23,28,37,44,49,54,55,59].
RQ7	Why should UUVs be used for surveys?	UUVs provide many advantages over traditional survey methods, such as flexibility, cost-effectiveness, and the ability to access areas that are difficult or dangerous for divers [31,32,56,62,64,65].
RQ8	How do UUV surveys present challenges?	UUV surveys can be challenging due to the need for specialized equipment and expertise, as well as the inability to operate in poorly lit or difficult-to-access underwater environments [60].

**Table 2:** Using existing surveys as references, mindmaps are compiled, comparisons are made, research approach models are applied, future trends are examined, and contributions are determined.

Research	Year	RQ1	RQ2	RQ3	RQ4	RQ5	RQ6	RQ7	RQ8
Al Guqhaiman et al. [7]	2021			*√*					
Wang et al. [20]	2022		*√*						
Zhang et al. [21]	2022		*√*						
Shi et al. [23]	2020		*√*				*√*		
Wu et al. [28]	2020	*√*	*√*				*√*		
Hong et al. [31]	2020	*√*		*√*		*√*		*√*	
Nakath et al. [32]	2022	*√*						*√*	
Karmozdi et al. [37]	2020		*√*				*√*		
Klein et al. [41]	2022	*√*							
Braginsky et al. [42]	2020	*√*							
Perea-Storm et al. [43]	2020		*√*						
Jiang et al. [44]	2022		*√*				*√*		
Yin et al. [49]	2022	*√*				*√*	*√*		
Sezgin et al. [52]	2022				*√*				
Hou et al. [54]	2023		*√*				*√*		
Neira et al. [55]	2021		*√*			*√*	*√*		
Luo et al. [56]	2021							*√*	
Lindsay et al. [59]	2022		*√*	*√*			*√*		
Purser et al. [60]	2022								*√*
Luo et al. [56]	2022			*√*					
Yan et al. [62]	2020				*√*	*√*		*√*	
Fang et al. [64]	2022				*√*	*√*		*√*	
Jiang et al. [65]	2023				*√*	*√*		*√*	

**Table 3:** List of acronyms.

Acronym	Definition	Acronym	Definition
USV	Unmanned Surface Vehicle	GPS	Global Positioning System
UAV	Unmanned Aerial Vehicle	ANS	Acoustic Navigation System
UUV	Unmanned Underwater Vehicle	VO	Visual Odometry
EC	Energy Consumption	UWSN	Underwater Wireless Sensor Network
TX	Tranceiver	ROV	Remotely Operated Vehicle
RX	Receiver	AWBC	Air–Water Boundaries Communication System
SS	Spherical Spreading	UCPS	Underwater Cyber–Physical System
BER	Bit Error Rate	IoUT	Internet of Underwater Things
SNR	Signal-to-Noise Ratio	CATM	Controversy-Adjudication-Based Trust Management
PID	Proportional Integral Derivative	MPC	Model-Predictive Control
SMC	Sliding Mode Control	AWBC	Air–Water Boundaries Communication System
AC	Adaptive Control	UCPS	Underwater Cyber–Physical System
AI	Artificial Intelligence	IoUT	Internet of Underwater Things
ML	Machine Learning	CATM	Controversy-Adjudication-Based Trust Management
KPI	Key Performance Indicators	CDV	Cross-Domain Vehicle
SoNAR	Sound Navigation and Ranging	DOF	Degrees of Freedom
LiDAR	Light Detection and Ranging	ITSM	Integral Terminal Sliding Mode
INS	Inertial Navigation System	FITSM	Fast Integral Terminal Sliding Mode
DVL	Doppler Velocity Log	AUV	Autonomous Underwater Vehicle
GNSS	Global Navigation Satellite System	DRL	Deep Reinforcement Learning
RGB	Red Green Blue	HSV	Hue Saturation Value
DVL	Doppler Velocity Log	UTM	Universal Transverse Mercator
WGS	World Geodetic System	RIS	Reconfigurable Intelligence Surface

**Table 4:** List of publishers and the number of publications surveyed.

Database	Number of Papers
IEEE Xplore	49
ScienceDirect-Elsevier	4
MDPI	7
SpringerLink	8
Hindawi	5
Wiley	4
Inder Science Online	1

**Table 5:** The definitional variable furnishes comprehensive insights into every symbol and unit employed to elucidate the equations within a cross-domain vehicle (CDV) motion system [22].

Symbol	Explanation
θ	The angle of pitch
$F_{f}$	The force exerted by the front hydrofoil
$I_{f}$	The distance between the front hydrofoil and the center of gravity
$I_{r}$	The distance between the rear hydrofoil and the center of gravity
$F_{r}$	The force applied by the rear hydrofoil
*P*	The thrust generated by the water jet propeller
*F*	The combined buoyancy and drag force of the CDV
*G*	The force of gravity
$F_{v}$	The force generated by the vertical propeller
*M*	The moment caused by buoyancy
α	The angle between *F* and the axial direction of the CDV
β	The angle formed between $F_{f}$ and the axial direction of the CDV
γ	The angle included between $F_{r}$ and the axial direction of the CDV
φ	The angle of roll
$U_{i,j}$	The velocity components in the *i* and *j* directions
$I_{v}$	The distance between the vertical propeller and the center of gravity
$J_{k}$	The moment around the *k* axis

The pitch angle (θ) of the cross-domain vehicle (CDV) induces lift forces ($F_{f}$) on the hull through the action of the front hydrofoil. The magnitudes of these forces are determined by considering the distances ($l_{f}$ and $l_{r}$) from the center of gravity to the front and rear hydrofoils, respectively. The angle β represents the included angle between the lift force ($F_{f}$) and the axis direction of the CDV. This angle is calculated through inverse trigonometric functions based on the lift and drag components of the front hydrofoil. A similar analysis can be performed for the lift force ($F_{r}$) generated by the rear hydrofoil on the hull. This interplay of pitch angles, hydrofoil forces, and geometric considerations contributes to the dynamic equilibrium and motion characteristics of the CDV in its operational environment.

**Table 6:** Summary of references regarding renewable energy sources that can be considered as alternative sustainable energy supplies for UUV operations [53].

Sustainable Energy Sources	The Form of Energy Generated	Types of Vehicles That Can Apply It
Hydrogen–Oxygen fuel cell	Heat–Electric energy	ROV, AUV
Photovoltaic energy	Heat–Electric energy	AUV, USV, and UG
Ocean wave power	Mechanical–Electrical energy	USV, AUV
Heat energy	Pressure–Electric energy	USV, AUV, and UG with profilling float
Marine current energy	Mechanical–Electric energy	UG, AUV

Abbreviations: ROV, remotely operated vehicle; AUV, autonomous underwater vehicle; USV, unmanned surface vehicle; UG, underwater glider.

**Table 7:** Summary of reference used to describe mission scenarios.

Research	Contribution	Outcome
Shen et al. [67]	Buoy transmitter relay	Cover communications for surface waters area
Qu et al. [9]	Underwater wireless acoustic communication	Cover long-distance transmission at sea depth
Al-Halafi et al. [10]	Underwater wireless optical communication	Cover short-range communication at sea depth
Gupta et al. [11]	Underwater wireless communication radio-waves	Cover communication at sea level
Page et al. [13,14]	Direct electrical system	A system that allows recharging for UUV
Luo et al. [29]	Air–water boundaries communication	Air–water communication link
Wang et al. [15,16,17]	Omnidirectional propulsion and dive system	A system that allows UUV free to move to all directions in underwater
Shi et al. [22]	Hydrojet propulsion and dive system	Allows the UUV to capable of operating in surface and underwater environments
Zhang et al. [21]	Undulating propulsion and dive system	UUV can move in an ideal fluid with a constant velocity
Saback et al. [24,68]	MPC algorithm	Capable to optimize control of UUV based on the prediction
Qiao et al. [25]	Sliding mode control algorithm	Considers simpler logical systematics but can withstand disturbances and uncertainties
Chu et al. [69]	Deep reinforcement learning control base	Control system based on the nervous system with the ability to make decisions based on training and past learning experience in recognizing the environment
Wolek et al. [30]	Use of fusion sensors	Make UUV have the ability to recognize the environment, detect the presence of underwater objects, or detect the presence of fellow UUV herds
Song et al. [34]	Use of optical sensors	Accurately used to recognize the environment based on image capture
Braginsky et al. [42]	Localization using DVL method	Can compute velocity and direction UUV using acoustic-beam
Perea-Strom et al. [43]	Localization using GPS–GNSS	UUV localization using GPS–GNSS, while currently only being able detect USV, will, in the future, be applicable for types of UUV after the discovery of air–water boundaries communication technology
Baik et al. [51,52,53]	Supply of power	Potential renewable energy based on fuel cell, solar, wind, wave, thermal, and tidal current energy

Reference was used to describe mission scenario.

**Table 8:** Identified issues and proposed solutions.

Identified Issues	Proposed Solutions
Cross-border communication [70,71]	Optimization can be achieved through collaborative mission management involving UAVs, USVs, and UUVs, utilizing underwater communication network infrastructure resources. Additionally, the use of surface buoys as relays, assisted by satellites, can help extend the coverage area.
Movement and dive system [16,21,22]	Optimization can be achieved by employing biorobotic mechanisms or vehicles inspired by living organisms. This approach is more efficient in generating propulsion and minimizing energy consumption.
Control system [24,25,68]	Optimization can be achieved through the implementation of adaptive control mechanisms, enabling vehicles to autonomously react to obstacles along the mission path and optimize routes based on predictions.
Sensing [30,34]	Optimization can be achieved by implementing a holistic sensing approach, wherein unmanned underwater vehicles can employ various types of sensors or diverse measurement methods in an integrated manner. This allows for a more comprehensive and profound understanding of the surrounding environment.
Localization [42,43]	Optimization can be achieved through passive underwater localization techniques that utilize the Doppler Velocity Log (DVL) sensor to ascertain the vehicle’s position in relation to the seafloor surface.
Supply energy [51,52,53]	Optimization can be accomplished by harnessing the potential renewable energy available in the vicinity of the operational area, while considering the ambient temperature of each model. This is essential as the storage capacity of batteries is influenced by ambient temperature.
Machine learning [82,83,84,85,86]	All the sub-technologies that support the operation of unmanned underwater vehicles can be optimized through the utilization of Machine Learning. This includes the optimization of the sensing system to accurately recognize underwater objects, avoid and prevent collisions, make predictions, and formulate measurable decisions, should similar challenges arise in the future.

The proposed solutions are expected to address common issues and challenges in the field of unmanned underwater vehicle research.

## Data Availability

Not applicable.
